# The mechanistic role of adipokine imbalance in airway inflammation, hyperresponsiveness, and remodeling in obesity-related asthma

**DOI:** 10.3389/fendo.2026.1894904

**Published:** 2026-09-25

**Authors:** Mingyue Ren, Guihua Song, Bingxue Zhang, Mengmeng Sun, Suping Yu, Yan Zhang

**Affiliations:** 1 Department of Pediatrics, The First Affiliated Hospital of Henan University of Chinese Medicine, Zhengzhou, China; 2 School of Pediatrics, Henan University of Chinese Medicine, Zhengzhou, China

**Keywords:** adipokines, airway hyperresponsiveness, airway inflammation, airway remodeling, obesity-related asthma

## Abstract

Obesity-related asthma (OBA) is a clinically important asthma phenotype characterized by poor disease control, increased exacerbation risk, and frequent corticosteroid insensitivity. Although obesity-related mechanical loading contributes to respiratory symptoms, it does not fully explain the inflammatory, functional, and structural features of this phenotype. This review examines adipokine dysregulation, particularly leptin excess and adiponectin insufficiency, as a biologically plausible axis linking systemic metabolic dysfunction to airway-relevant pathology. Epidemiological, animal, and airway-cellular evidence supports adipokine involvement of variable strength in three interconnected domains, including immune amplification through context-dependent macrophage and NLRP3 inflammasome activation, airway hyperresponsiveness (AHR) through inflammatory, neural bronchomotor, and airway smooth muscle (ASM) pathways, and remodeling through epithelial repair failure, ASM proliferation, peribronchial adipose tissue (PBAT) and airway-associated adipose tissue (AAAT) adipose-airway crosstalk, fibroblast activation, and extracellular matrix (ECM) deposition. A spatial-temporal model is proposed in which systemic endocrine adipokine signals may be complemented by local adipose-airway paracrine effects, while persistent metabolic inflammation may shift disease expression from a potentially reversible inflammatory and hyperresponsive state toward remodeling-predominant pathology. Clinically, adipokines are best viewed as candidate stratification markers integrated with metabolic, inflammatory, functional, and structural readouts, and metabolic interventions should be interpreted as phenotype-directed interventions with potential disease-modifying relevance and biomarker endpoints. The translational value of this framework lies in guiding airway-focused validation, longitudinal phenotyping, and mechanistic intervention trials that can distinguish weight-loss effects from direct metabolic-inflammatory airway modulation.

## 1 Introduction

Obesity-related asthma (OBA) represents a clinically distinct phenotype characterized by suboptimal disease control, elevated exacerbation risk, and frequent corticosteroid resistance (1). In 1,113 adults with active asthma, obesity was associated with poorer asthma-specific quality of life and control, and higher odds of asthma-related hospitalization after confounders adjustment (2). Globally, over 890 million adults have obesity, which confers about 51% higher asthma risk (3, 4). In GBD 2021, High Body Mass Index (BMI) accounted for 14.9% of global asthma disability-adjusted life years (DALYs) and was the leading risk factor for asthma-related DALYs in high-sociodemographic-index settings (5). Studies have shown that adults who are overweight, obese, or transition from non-obese to obese status have significantly increased asthma risk, with the relationship being nonlinear and risk rising sharply above specific BMI thresholds such as greater than 29.9 kg/m² (6). While mechanical consequences of obesity, including reduced lung volumes and increased airway wall loading, contribute to disease burden, they may not fully explain the inflammatory, functional, and treatment-response features of this phenotype (7–12). Accumulating evidence points to adipokine dysregulation as one candidate intermediary axis linking systemic metabolic dysfunction to airway-relevant outcomes (13, 14).

The biological plausibility of an adipokine-mediated mechanism is supported by several converging lines of mechanistic and clinical evidence. In mice, high-fat diet (HFD)-induced obesity and insulin resistance can precipitate pulmonary fibrosis and airway hyperresponsiveness (AHR) through TGF-β_1_ signaling and obesity-associated AHR can also arise through inflammasome-linked ILC3/IL-17A pathways without classical adaptive immune activation (15, 16). In humans, reduced corticosteroid responsiveness in obesity is supported by original clinical and mechanistic studies rather than by review-level evidence alone. Pooled randomized controller-trial analyses found that increasing BMI was associated with reduced response to inhaled beclomethasone (8). CAMP trial data showed attenuated budesonide responses in overweight or obese children (9). Obese adults with asthma showed smaller FEV_1_ and sputum eosinophil responses after corticosteroid treatment, while Asthma Clinical Research Network data found smaller FeNO or lung-function improvements after ICS-containing regimens in overweight/obese subjects (10, 11). Complementary ex vivo PBMC/BAL-cell studies demonstrated reduced dexamethasone-induced MKP-1 expression as BMI increased (12). Obesity-associated metabolic states are often accompanied by adipokine imbalance, including relatively higher leptin and lower adiponectin, creating a tractable axis for connecting systemic metabolic dysfunction to airway outcomes (13). High-fat feeding triggers insulin resistance, adipose-tissue macrophage accumulation (17–19), and systemic low-grade inflammation, creating a metabolic-inflammatory milieu relevant to AHR. The specificity of the adipokine contribution is more directly supported by mechanistic studies showing that leptin can promote allergic airway inflammation, whereas adiponectin receptor (AdipoR) activation attenuates allergen-induced airway pathology, findings that support a directional role for individual adipokines beyond the confounding metabolic milieu of high-fat feeding (20, 21). Dixon et al. showed that bariatric surgery improved AHR, asthma control, and inflammatory measures, and van Huisstede et al. reported improvements in asthma control, lung function, and bronchial or systemic inflammatory measures in morbidly obese asthmatic subjects (22, 23). These intervention indicate that modifiable obesity-associated factors shape airway outcomes and support incorporating metabolic and adipokine endpoints in future metabolic-intervention cohorts.

This review focuses on adipokine imbalance as one contributing mechanism in OBA, examining the biological basis of altered adipokine profiles, immune mechanisms of airway inflammation, AHR pathogenesis, and airway remodeling. To link systemic metabolic dysfunction with local airway pathology, it also incorporates a spatial perspective on the transition from endocrine to paracrine signaling. Given an evidence base that spans animal models, *in vitro* studies, epidemiological associations, and human mechanistic data, claims are presented in a tiered manner matched to their translational endpoint. Animal and cellular findings define mechanistically plausible pathways, whereas human associative or cross sectional findings guide phenotype interpretation and prospective validation. The objective is to provide an integrated mechanistic framework that supports cautious phenotype interpretation while highlighting testable entry points for biomarker development, metabolic intervention studies, and pathway-enriched clinical trials in OBA.

## 2 The biological basis of adipokine imbalance and its changes in OBA

### 2.1 Adipose tissue as an active endocrine and immunomodulatory organ

Adipose tissue functions not merely as a depot for energy storage but as a metabolically active endocrine organ capable of influencing systemic immune responses through the secretion of a diverse repertoire of bioactive peptides collectively termed adipokines (24). Under conditions of obesity, adipocyte hypertrophy and tissue hypoxia can disrupt the homeostatic balance of adipokine secretion, resulting in augmented release of pro-inflammatory mediators alongside a reciprocal reduction in adiponectin, as shown most directly in human adipocyte hypoxia experiments (25). Adipose-tissue investigations show that obesity is accompanied by macrophage accumulation in white adipose tissue and increased expression of inflammatory mediators such as TNF-α, iNOS, and IL-6 (18, 19). Human adipocyte experiments further indicate that hypoxia can increase leptin, VEGF, and IL-6 release and suppress adiponectin secretion (25). These findings define a self-amplifying adipose inflammatory loop that can contribute to systemic metabolic inflammation and provide a mechanistic entry point for studying airway consequences in OBA.

However, the association between lipid factor imbalance and the pathological state of asthma is influenced by factors such as disease severity, body weight, and therapeutic interventions. A systematic review and meta-analysis demonstrated that serum leptin concentrations are elevated in patients with asthma relative to non-asthmatic controls, while clinical studies continue to examine leptin-related markers in severe asthma populations (26, 27). Conversely, adiponectin levels are often reported to be lower in adult or obesity-dominant asthma contexts, although age- and phenotype-specific heterogeneity remains important (26). These population-level associations provide the foundational epidemiological rationale for investigating adipokine dysregulation as a mechanistically relevant contributor to asthma pathogenesis.

Adipokine dysregulation does not operate in metabolic isolation. In many obese individuals, insulin resistance frequently co-segregates with adipokine imbalance and may contribute to pro-inflammatory signaling (28). Because their downstream effects on airway inflammation partially overlap, disentangling the specific contribution of each perturbation in human studies remains methodologically challenging. This review focuses on the adipokine axis as a biologically distinct and experimentally tractable component of OBA. Clarifying the independent and combined contributions of these co-occurring metabolic abnormalities could improve metabolic-airway phenotyping and help identify patients most likely to benefit from targeted metabolic interventions.

### 2.2 Leptin excess and the evidence for its pro-inflammatory role

Leptin is a 16 kDa protein hormone secreted predominantly by white adipocytes, and its circulating concentrations rise in direct proportion to adipose tissue mass, resulting in chronic hyperleptinemia in many individuals with obesity (29). Experimental and airway-relevant cellular evidence supports a pro-inflammatory, cell context-dependent effect across immune and structural compartments. In ovalbumin-sensitized mice, exogenous leptin augmented allergen-induced AHR and IgE without independently inducing AHR in saline-challenged animals (30). Leptin enhanced Th2/ILC2 responses in allergic airway models, promoted unfolded-protein-response signaling in Th2 cells, increased IL-13-induced MUC5AC production in human bronchial epithelial cells, and aggravated house dust mite-induced airway inflammation partly through macrophage necroptosis (20, 31–33). In human immune cells, leptin can activate basophils by promoting survival, migration, degranulation, and cytokine release, and enhance eosinophil survival, chemokinesis, migration, and cytokine release (34–36). In structural cells, leptin increased airway epithelial ICAM-1 expression and cytokine synthesis, while airway smooth-muscle studies showed VEGF release with context-dependent proliferation effects (37–39). These findings indicate that leptin’s airway effects are better mapped by cell type, inflammatory state, and receptor responsiveness than a single uniform pathway. Translational evidence in humans, while more limited, offers complementary support. In the French EGEA cohort, mediation analysis suggested that leptin accounts for a meaningful proportion of the statistical association between adiposity and persistent asthma (14). This observation provides supportive population-level evidence and a rational basis for prospective adipokine profiling and airway-sample validation. Overall, the available evidence supports leptin excess as a biologically plausible and directionally consistent contributor to obesity-related airway disease and highlights the need for studies that quantify its contribution using paired systemic, airway, and cellular readouts.

The relationship between leptin and airway disease is also modified by sex and reproductive stage. Circulating leptin concentrations are often higher in females than in males at comparable adiposity, a pattern that may reflect sex-related hormonal and adipose-tissue differences (40). In a population-based adult study, serum leptin was associated with current asthma more strongly in women, particularly premenopausal women, and adjustment for leptin only partly accounted for the BMI-asthma association (41). Reviews and recent real-world severe-asthma data further show clinically important sex differences in asthma burden and obesity distribution (42, 43). These sex-related differences have important implications for the interpretation of studies that do not stratify analyses by sex, and underscore the necessity of sex-disaggregated reporting in future adipokine asthma research.

### 2.3 Adiponectin deficiency, pediatric-adult divergence, and endotype-specific heterogeneity

Adiponectin exerts predominantly anti-inflammatory and bronchoprotective effects under physiological conditions, with the most direct asthma-relevant support coming from mouse intervention and deficiency studies involving AdipoR/AMPK-related signaling. AdipoR agonism alleviated ovalbumin-induced airway inflammation through AMPK-related signaling (21). Exogenous adiponectin attenuated ovalbumin-induced airway inflammation and AHR (44), adiponectin deficiency increased allergic airway inflammation and pulmonary vascular remodeling, and adiponectin treatment reduced inflammatory and oxidative injury in an obesity-related asthma mouse model partly through AMPK signaling (45, 46). These data position the AdipoR/AMPK pathway as a bronchoprotective signaling axis that can be linked to airway and sputum-based translational endpoints. In adults with obesity-dominant asthma, lower adiponectin levels are consistent with loss of an anti-inflammatory metabolic constraint, whereas airway AdipoR abundance and AMPK activity remain important translational endpoints derived primarily from mechanistic studies (21, 26, 46).

Epidemiological data, however, introduce a complicating paradox in which Mendelian randomization analyses have not confirmed a robust causal relationship between genetically determined adiponectin levels and asthma risk, whereas pediatric cohorts of overweight or obese children with asthma show adipokine patterns that may differ from the adult obesity-dominant model (47, 48). This pattern is better understood in the context of pediatric clinical and immune-endotype heterogeneity rather than as a contradiction of the adult OBA model. Pediatric asthma is commonly shaped by allergic sensitization and viral-triggered exacerbation risk (49, 50), whereas adult OBA more frequently includes metabolic dysfunction, non-T2 inflammation, and cumulative structural airway injury. Accordingly, in pediatric asthma, obesity may act more often as a disease modifier that increases symptom burden, severity, and treatment difficulty, rather than as evidence that metabolic inflammation is the principal initiating mechanism in all cases (51, 52).

Developmental biology further supports this boundary. Alveolarization continues beyond early childhood and lung-resident macrophage populations undergo developmentally regulated postnatal maturation (53, 54). Single-cell, immune-cell, and spatial atlases of the developing human lung show that epithelial, stromal, endothelial, and immune compartments occupy temporally regulated developmental niches, while IL-1β-producing myeloid cells can localize near epithelial progenitors and influence epithelial cell fate (55, 56). These developmental features support a pediatric-specific framework in which adipokines may alter inflammatory thresholds, epithelial repair responses, or cytokine profiles within an airway still undergoing immune and structural maturation. Pediatric obese asthma is therefore best approached as an endotype-stratified condition in which allergic sensitization and viral-exacerbation tendency are considered alongside pubertal status, sex, fat distribution, and metabolic dysfunction rather than BMI alone.

This pediatric-adult distinction also highlights several variables that may shape adiponectin measurements and signaling responses, including age, adiposity distribution, insulin resistance, corticosteroid exposure, allergic inflammatory burden, and pediatric adipokine-profile heterogeneity (13, 26, 28, 48). Circulating adiponectin concentration may not mirror airway AdipoR abundance or downstream AMPK responsiveness, a pattern conceptually analogous to systemic adiponectin resistance described in obesity (57). Future studies that integrate systemic adipokine profiling with airway epithelial, immune-cell, and airway smooth-muscle (ASM) measurements of AdipoR expression and AMPK activity, while stratifying cohorts by age, puberty, sex, atopy, T2 biomarkers, and metabolic phenotype, could help distinguish protective adiponectin signaling from receptor-level hyporesponsiveness.

Beyond leptin and adiponectin, several additional adipokines have been implicated as supplementary modulators of asthma pathology. Within the BIOAIR cohort, adipokine patterns, including chemerin, appeared to be influenced by sex, body weight, and oral corticosteroid exposure, underscoring the need to interpret these mediators within clinical and treatment contexts (13). Resistin has been associated with asthma risk in epidemiological analyses, and an elevated resistin-to-adiponectin ratio correlates negatively with FEV_1_ (58). These molecules can be conceptualized as components of a broader pro-inflammatory adipokine network that may refine inflammatory phenotyping when analyzed alongside leptin and adiponectin. In addition to these circulating mediators, local adipose depots may also contribute to airway signaling. Airway-associated adipose tissue (AAAT) is a broad anatomical designation for adipose tissue located within or immediately adjacent to the airway wall (59). Peribronchial adipose tissue (PBAT) refers to adipose tissue surrounding the bronchial outer wall, which represents a plausible local adipose-airway interface within AAAT. Human post-mortem studies have identified adipose tissue in the outer wall of large airways, with adipose area correlating with BMI, airway wall thickness, and inflammatory-cell density, thereby supporting the presence and disease relevance of PBAT (60). These findings suggest that wall-adjacent adipose tissue, best exemplified by PBAT, may provide local paracrine adipokine signals in addition to systemic endocrine spillover. This dual systemic-local architecture is summarized in **Figure 1**.

**Figure 1:**
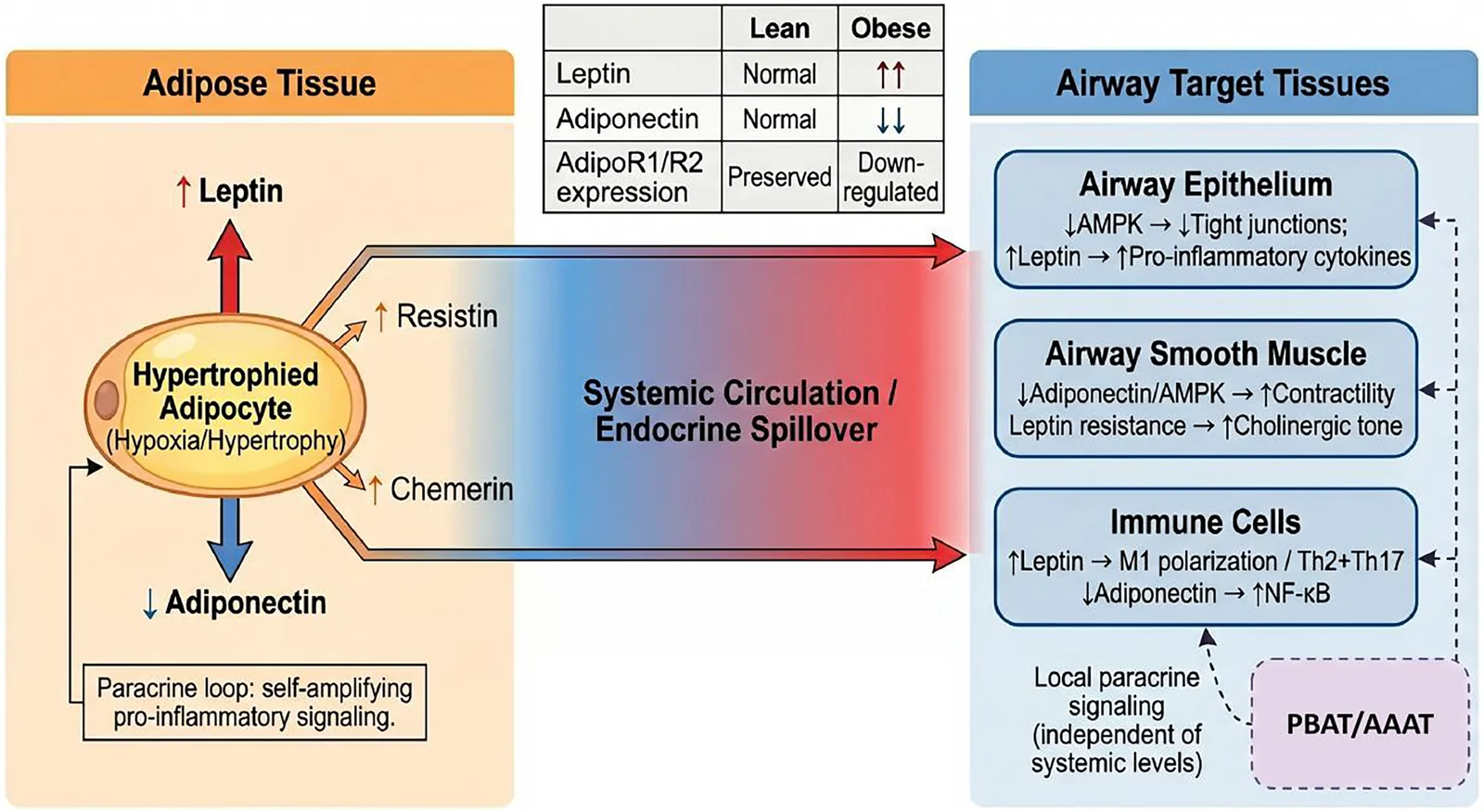
The adipokine imbalance axis linking obesity to airway pathology. Under conditions of obesity, adipocyte hypertrophy and hypoxia shift the adipokine secretory profile toward pro-inflammatory excess (leptin↑, resistin↑, chemerin↑) and anti-inflammatory insufficiency (adiponectin↓), establishing a self-amplifying inflammatory loop within adipose tissue. Systemic endocrine spillover may expose airway epithelial cells, airway smooth muscle, and resident immune cells to sustained adipokine dysregulation, while wall-adjacent adipose tissue may provide local paracrine adipokine signals to airway wall tissues. PBAT, peribronchial adipose tissue surrounding the bronchial outer wall; AAAT, airway-associated adipose tissue; AMPK, AMP-activated protein kinase; NF-κB, nuclear factor κB; AdipoR, adiponectin receptor.

## 3 Mechanisms of adipokine-driven airway inflammation

### 3.1 Adipokine-driven modulation of innate immune responses

The innate immune system constitutes the principal initiator of airway inflammation in OBA, and adipokine dysregulation can influence innate immune programs in OBA through macrophage-, neutrophil-, and eosinophil-linked pathways. These pathways are best organized as three interconnected cellular interfaces: leptin/obR- and adiponectin-linked macrophage activation programs, leptin-associated neutrophil recruitment and survival, and adipokine-sensitive eosinophil or fibroblast responses in allergic or mixed disease.

The macrophage axis provides the clearest experimental bridge between adipokine imbalance and non-T2 airway inflammation. Consistent with current airway immunology, these cells are better framed as plastic, marker-defined activation states rather than a fixed M1/M2 identity (61–63). In obesity-related neutrophilic airway inflammation models, leptin/obR signaling favored inflammatory macrophage activation programs that earlier studies often labeled “M1 polarization”, and increased Th1/Th17-related inflammatory cytokines, while obR blockade or deficiency attenuated these responses (64). Macrophage experiments further demonstrated that leptin can activate the JAK2/STAT3 signaling pathway and enhance the expression of CCL3, CCL4, and CCL5 in cultured mouse macrophages (65). These leptin/obR-driven inflammatory macrophage programs activity also increased CXCL2 production and neutrophil recruitment in airway-focused experimental systems, and obese patients with asthma showed higher serum leptin levels and sputum signatures of inflammatory macrophage activation than non-obese patients with asthma (64). These findings align with non-T2, neutrophil-predominant inflammation in obese asthma and identify the leptin-macrophage axis as a candidate pathway for sputum- and bronchoalveolar lavage-based phenotyping studies.

Adiponectin, in its physiological state, can counterbalance selected pro-inflammatory effects by suppressing macrophage cytokine signaling. In human macrophages, adiponectin pretreatment inhibited LPS- or TNF-α-induced IκB, JNK, and p38 MAPK phosphorylation and induced several anti-inflammatory regulatory proteins, while macrophage inflammasome studies indicate that globular adiponectin can suppress LPS-primed inflammasome activation through AMPK-linked autophagy mechanisms (66, 67). Under obese conditions, reduced adiponectin secretion and possible receptor-level hyporesponsiveness may weaken this homeostatic brake, supporting consideration of macrophage adiponectin signaling within broader immune-regulatory frameworks for OBA (68).

The neutrophil interface should be separated from the macrophage axis because the direct effects of leptin on human neutrophils are context dependent. Human neutrophil studies indicate that leptin responsiveness varies with concentration and activation state, including reports of chemotaxis or oxidative responses as well as minimal effects at physiological concentrations (69, 70). Pediatric neutrophil experiments show leptin-mediated survival through ERK/NF-κB-dependent pathways, whereas *in vivo* work indicates that leptin can promote neutrophil migration through TNF-α/CXCL1-dependent pathways (71, 72). In airway-specific obesity models, leptin/obR-driven inflammatory macrophage programs increased CXCL2-dependent neutrophil recruitment, and a recent HDM model showed that high-fat diet-induced obesity intensified lung inflammation with increased neutrophilic infiltration and non-Th2 cytokines, including IL-17, TNF-α, TGF-β, and IL-1β (64, 73). These findings support a neutrophil-centered inflammatory route in metabolically enriched or mixed OBA, but they also justify avoiding a simple statement that leptin uniformly activates neutrophils in all contexts.

In allergic or mixed obese asthma, eosinophils and airway fibroblasts provide a separate adipokine-sensitive interface. Human eosinophils express functional leptin receptors and adiponectin receptors, indicating direct responsiveness to local or systemic adipokine signals (35, 36, 74). Leptin can enhance eosinophil chemokinesis, prime chemokine-driven migration, promote adhesion-molecule changes, induce IL-1β, IL-6, IL-8, GRO-α, and MCP-1 release, and prolong eosinophil survival in experimental systems (35, 36), whereas adiponectin can attenuate eotaxin-induced eosinophil adhesion and chemotaxis in a concentration-dependent manner (74). In parallel, leptin can potentiate IL-13-driven epithelial mucin responses and airway fibroblast eotaxin production, linking adipokine excess to mucus biology and tissue eosinophil retention (32, 75). Experimental chronic allergic airway models further suggest that leptin may increase fibrosis-related remodeling markers (76). This evidence supports a focused downstream interpretation that macrophage- and granulocyte-centered adipokine signaling may contribute to AHR and remodeling when it converges with epithelial chemokine production, IL-13-rich mucus responses, or fibroblast-associated eosinophil recruitment, rather than by a single uniform inflammatory route across all OBA endotypes.

Overall, animal models, *in vitro* granulocyte and macrophage studies, and translational human observations support an adipokine-innate immune axis in OBA, with particularly tractable macrophage/neutrophil and eosinophil/fibroblast interfaces.

### 3.2 Phenotype-conditional mechanisms in adipokine dysregulation and adaptive immune responses

The relationship between adipokine dysregulation and adaptive immunity in OBA is characterized by substantial complexity and apparent internal tension in the existing literature, which can be resolved by recognizing that distinct immunological mechanisms predominate in different disease subphenotypes.

In allergic, T2-predominant OBA, leptin may function as an amplifier of effector lymphocyte responses. Experimental work indicates that leptin enhances Th2 and ILC2 responses in allergic airway disease models and can promote proliferation, survival, and cytokine production in pro-allergic lymphocytes through mTORC1, MAPK, and STAT3-related pathways (31). Additional clinical immune-profiling evidence links leptin to Th17/Treg imbalance in allergic asthma severity, while general T-cell metabolic evidence from experimental systems shows that leptin can promote glycolytic reprogramming and effector T-cell differentiation (77, 78). Together, these findings support leptin as a candidate amplifier of adaptive immune activity in allergic or mixed OBA, especially when adipokine profiling is combined with airway-localized and phenotype-stratified immune readouts.

In non-allergic or non-T2 obese asthma, the contribution of adipokine dysregulation to adaptive immune amplification may be less direct. HFD-induced AHR can occur in experimental models without classical adaptive immune activation, involving macrophage-derived IL-1β, expansion of ILC3, and downstream IL-17A production (16). This finding helps define the boundary of adipokine-adaptive immune interactions by showing that, in some obese asthma contexts, airway dysfunction may be driven predominantly by innate/type-3 inflammatory programs rather than by conventional antigen-driven adaptive immunity. Clinically, this distinction is relevant because obese-asthma cohorts show greater neutrophilic inflammation and weaker corticosteroid responses, while human ILC3 experiments indicate that IL-1β-induced CXCL8/CXCL1 production can be insensitive to dexamethasone through aberrant glucocorticoid receptor phosphorylation (10, 79). Together, these findings support a phenotype-conditional framework in which allergic or T2-predominant OBA is more likely to involve leptin-associated adaptive immune amplification, whereas non-allergic or non-T2 OBA may show weaker or more indirect adaptive immune involvement and greater reliance on innate/type-3 inflammatory programs such as the IL-1β–ILC3–IL-17A axis.

### 3.3 Adipokine effects on the airway epithelial barrier and innate inflammatory amplification

#### 3.3.1 Epithelial barrier dysfunction and impaired regenerative capacity

Airway epithelial cells provide a relevant structural interface for adipokine-related signaling because they serve as both the main barrier against inhaled stimuli and an active participant in inflammatory amplification (80). Human bronchial epithelial-cell studies show that asthmatic epithelium can exhibit reduced tight-junction integrity, lower transepithelial resistance, increased paracellular flux, and disrupted occludin/ZO-1 organization, while oxidative stress can increase epithelial permeability through TRPM2-PLCγ1-PKCα-dependent tight-junction disruption (81, 82). The decline in adiponectin may plausibly reduce AMPK-mediated antioxidant protection in airway epithelial cells, linking adiponectin deficiency to oxidative epithelial stress in a barrier-relevant context (46, 82). Simultaneously, the loss of adiponectin’s anti-inflammatory signaling may attenuate epithelial regenerative capacity following injury (83). Leptin, conversely, can enhance pro-inflammatory cytokine production by airway epithelial cells, potentially destabilizing the barrier microenvironment (37). Together, these epithelial data suggest that adipokine imbalance may regulate epithelial barrier dysfunction and impaired regenerative capacity in OBA, with adiponectin insufficiency linked to weakened antioxidant and repair-supporting epithelial responses and leptin excess linked to a pro-inflammatory epithelial microenvironment.

#### 3.3.2 Mucus hypersecretion and NLRP3 inflammasome-mediated inflammatory amplification

Leptin has been shown to enhance IL-13-induced MUC5AC production and secretion in human bronchial epithelial cells, indicating that hyperleptinemia may potentiate Th2 cytokine-driven mucin production (32). Separately, IL-33-induced models in leptin-deficient obese mice suggest that endogenous leptin contributes to goblet cell metaplasia and MUC5AC-related responses (84). Therefore, leptin plays a crucial regulatory role in mucous secretion.

Concurrently, metabolic dysregulation characteristic of obesity can activate inflammasome-related innate immune pathways through several experimentally defined stimuli in macrophage, adipose-tissue, or broader metabolic-inflammation systems. Saturated fatty acids such as palmitate induce NLRP3-ASC inflammasome activation and IL-1β/IL-18 production in macrophage systems, obesity-associated ceramide can activate NLRP3 signaling in adipose tissue and macrophages, and cholesterol crystals can trigger NLRP3-dependent IL-1 family cytokine release through lysosomal injury pathways (85–88). In airway-relevant but non-obesity-specific contexts, mixed eosinophilic/neutrophilic asthma models also support a role for epithelial NLRP3-mediated pyroptosis in AHR (89). Together, these mechanistic data support that metabolic danger signals may amplify IL-1β/IL-18 signaling and non-T2 inflammation in metabolically enriched asthma phenotypes, thereby extending epithelial injury and mucus dysregulation into a broader inflammatory microenvironment. Importantly, NLRP3 activation in this context may arise, at least in part, from metabolic stimuli intrinsic to the obese state in addition to classical pathogen-associated triggers, identifying it as a mechanistically distinct inflammatory amplifier in OBA.

Taken together, adipokine-related epithelial barrier disruption, secretory remodeling, and metabolic inflammasome activation define an inflammatory epithelial microenvironment that may prime the airway for exaggerated bronchoconstrictive responses.

## 4 Mechanisms of adipokine-driven AHR

### 4.1 Pathological significance of AHR in OBA

AHR represents a defining functional abnormality of asthma, reflecting an exaggerated bronchoconstrictive response to stimuli (90). In OBA, AHR appears to differ from that in classical atopic disease in at least some patients, because mechanical factors such as reduced functional residual capacity and central adiposity-related airway wall loading contribute to airway narrowing but do not fully account for corticosteroid-insensitive AHR, which may instead arise from the convergence of ASM dysfunction, persistent innate inflammatory activation, and impaired neural regulation of bronchomotor tone (91, 92). Experimental evidence supports this interpretation, showing that in HFD-induced models, obesity-linked IL-1β/ILC3/IL-17A inflammatory pathways can promote glucocorticoid-resistant AHR independently of classical adaptive immune activation and through pathways distinct from those targeted by conventional inhaled corticosteroids (16). Airway-relevant mixed-pattern asthma models further support NLRP3-linked epithelial pyroptosis in AHR outside an obesity-specific context (89), while metabolic inflammasome stimuli provide plausible upstream triggers for this inflammatory milieu (85–88). Together, these findings position AHR in OBA as a metabolically conditioned functional abnormality rather than a purely mechanical consequence of obesity.

### 4.2 Direct and indirect effects of adipokines on ASM function

Adipokines may modulate ASM contractility through direct receptor-mediated effects and indirect neural and inflammatory intermediaries. With respect to direct effects, the evidence for leptin-induced ASM contraction is heterogeneous across *in vitro* systems, so any direct contractile contribution remains uncertain (38, 93). By contrast, animal neurophysiological evidence support a neural modulatory role. Specifically, under non-obese conditions, leptin signaling through receptors on parasympathetic cholinergic nervous attenuates cholinergic tone and exerts tonic bronchodilation (94). In chronic obesity, however, progressive leptin resistance, arising from receptor desensitization and downstream signaling impairment analogous to hypothalamic leptin resistance, may disrupt this neural regulatory axis, leading to disinhibited parasympathetic activity, elevated cholinergic tone, and consequent enhancement of stimulus-evoked bronchoconstriction (94). This neural mechanism provides a parsimonious explanation for the heightened bronchomotor reactivity characteristic of obese asthma that is independent of airway wall inflammatory cell infiltration.

Adiponectin provides a complementary bronchoprotective axis. In obese asthma models, reduced adiponectin may weaken AMPK-mediated protection and increase bronchoconstrictive responses (46). Because this evidence remains largely animal-model based, the adiponectin-AMPK pathway remains a candidate modifier of ASM-related bronchomotor responsiveness that requires airway-focused human validation.

### 4.3 Reciprocal amplification between the inflammatory microenvironment and AHR

Obesity-associated AHR has been linked to systemic inflammation and altered inflammatory adipokines, suggesting that AHR in OBA may be sustained by metabolic-inflammatory signals rather than by adipokine imbalance alone (95). Experimental neutralization of TNF-α or depletion of alveolar macrophages in animal models significantly attenuates AHR, supporting a functional contribution of macrophage-derived inflammatory signaling to bronchial hyperreactivity (96). Conversely, recurrent bronchospasm may expose airway epithelial cells to mechanical shear and compressive stress. Cellular studies show that compressive stress can induce an asthma-like gene signature in human bronchial epithelial cells, including IL-33, TSLP, and CXCL8 induction, and that mechanically stressed bronchial epithelium can release TGF-β_2_ and endothelin mediators that stimulate fibrotic protein synthesis in fibroblast reporter systems (97, 98). Together, these findings support a reciprocal circuit in which obesity-related inflammation increase AHR, while repeated bronchoconstriction feeds back on the epithelium to promote stress and profibrotic programs. Because metabolic dysfunction can maintain inflammatory tone beyond episodic allergen exposure, this circuit provides a mechanistic bridge between metabolic inflammation, AHR persistence, and corticosteroid-insensitive disease activity in OBA.

### 4.4 An integrated tripartite model of AHR

Synthesizing the mechanistic evidence reviewed above, AHR in OBA can be conceptualized as arising from the convergent contribution of three partially independent axes, which differ materially in the strength and directness of available evidence. The first and currently strongest experimental axis comprises NLRP3 inflammasome activation and IL-17A-mediated persistent innate airway inflammation. The second axis involves potentially attenuated AMPK-mediated ASM protection in the setting of adiponectin deficiency. The third axis encompasses impaired neural bronchomotor regulation arising from obesity-induced leptin resistance.

Presenting these three axes as a convergent model does not imply equivalent evidential weight across all components. The relative contribution of each axis is likely to vary across individuals according to phenotypic characteristics, including adiposity distribution, degree of leptin resistance, atopic status, and concurrent metabolic comorbidities. The tripartite convergence of these axes, their differential evidential weightings, and their bidirectional interactions with the local inflammatory microenvironment are synthesized schematically in **Figure 2**.

**Figure 2:**
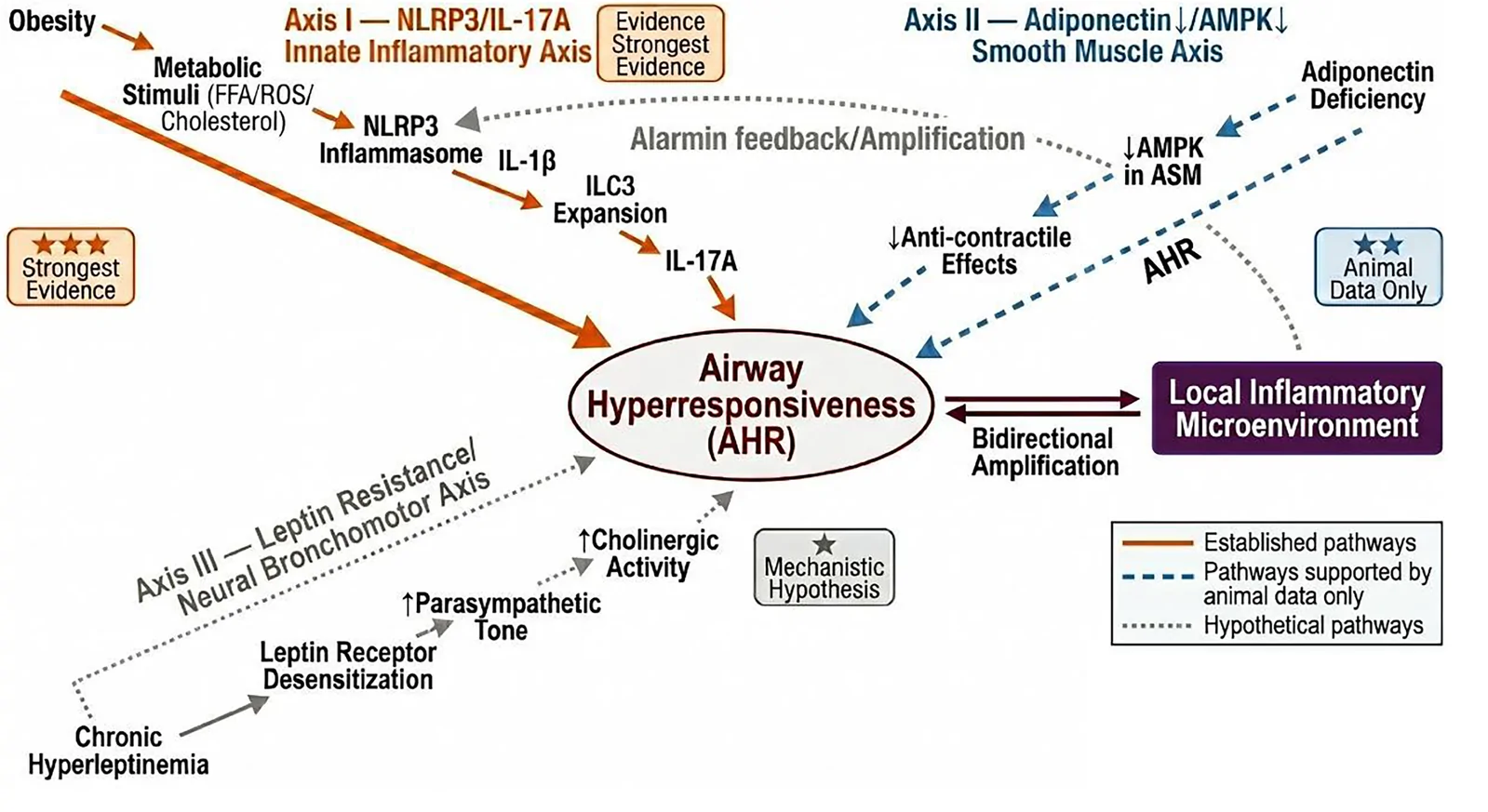
The integrated tripartite model of AHR in OBA. Three mechanistically distinct axes converge on AHR with differential evidential support. Bidirectional amplification between AHR and the local inflammatory microenvironment is shown for Axes I and II; Axis III highlights a neural bronchomotor pathway that can be prioritized for physiological testing. Line types reflect evidence quality as indicated in the legend. ASM, airway smooth muscle; FFA, free fatty acids; ROS, reactive oxygen species; ILC3, group 3 innate lymphoid cells; AHR, airway hyperresponsiveness.

## 5 Mechanisms of adipokine-driven airway remodeling

### 5.1 Local adipose-airway crosstalk involving PBAT and AAAT within the airway microenvironment

In evaluating adipokine-related mechanisms of airway remodeling, systemic endocrine concentrations should be distinguished from local concentration gradients within the airway microenvironment. Histologic studies have identified adipose tissue in the outer wall of large airways, and adipose tissue area correlates with BMI, airway wall thickness, and inflammatory cell densities (60). Additional experimental and clinical observations involving airway-associated adipose accumulation, leptin-driven airway fibroblast eotaxin responses, and asthma-linked resistin treatment-response associations further support the broader plausibility that adipose-related signals can influence airway tissue biology (59, 75, 99). In addition, human airway studies show that leptin can augment IL-13-induced eotaxin production in airway fibroblasts, whereas adipocyte-fibroblast coculture studies indicate that obesity-related adipocyte signals can promote fibroblast-to-myofibroblast transition through TGF-β1-centered pathways (75, 100). These findings position local adipose-airway crosstalk as a plausible contributor to fibroblast activation and structural remodeling in metabolically enriched asthma. Accordingly, the PBAT/AAAT axis can be tested by pairing systemic adipokine measurements with airway-wall or depot-specific adipokine profiling, airway imaging, biopsy-based remodeling endpoints, and patient-derived stromal-cell assays. This strategy can clarify whether local adipose-airway communication tracks with remodeling progression beyond circulating adipokine concentrations alone.

### 5.2 Leptin/LepR signaling at the inflammatory-remodeling interface

Airway remodeling constitutes a defining pathological feature of asthma, encompassing a constellation of persistent structural alterations that include subepithelial fibrosis, excessive extracellular matrix (ECM) deposition, ASM hyperplasia and hypertrophy, goblet cell metaplasia, and neovascularization (101, 102). HFD-induced insulin resistance promotes pulmonary fibrosis and AHR through TGF-β_1_ signaling in mice (15), airway-associated adipose tissue in humans correlates with BMI, airway wall thickness, and inflammatory-cell density (60), and obese-adipocyte/fibroblast coculture promotes myofibroblast transition and collagen deposition through TGF-β_1_-centered signaling (100). Together, these findings link metabolic dysfunction to structural airway change and identify TGF-β-related fibroblast activity as a candidate remodeling interface in OBA.

Leptin may influence this remodeling interface in a stage- and compartment-sensitive manner (103). During active airway inflammation and AHR, systemic hyperleptinemia with preserved receptor responsiveness can amplify inflammatory signaling through immune, epithelial, and neural pathways: leptin augments allergen-induced AHR and IgE responses in sensitized mice, enhances Th2/ILC2 responses, promotes epithelial MUC5AC production, activates macrophage-linked inflammatory programs, and regulates parasympathetic bronchomotor tone (30–33, 64, 94). Together, these studies define immune, epithelial, and neural interfaces through which leptin excess may influence inflammatory states that precede or accompany remodeling in OBA. In asthmatic airway epithelium, reduced leptin and leptin receptor expression has been associated with increased reticular basement membrane thickness and epithelial TGF-β expression; *in vitro*, TGF-β decreases leptin receptor expression, whereas leptin reduces spontaneous TGF-β release and increases epithelial proliferation (104). These observations support a cautious, testable tissue-level interpretation in which preserved leptin signaling is linked to inflammatory amplification, whereas attenuated local LepR signaling may be associated with TGF-β-related fibrotic activity, a pattern that requires longitudinal validation in OBA.

### 5.3 Adipokine-induced airway epithelial injury and aberrant repair

The airway epithelium is the principal interface with the external environment, and orderly post-injury repair determines whether inflammation resolves or progresses to remodeling (105). Human bronchial epithelial experiments show that adiponectin supports proliferation, wound repair, and apoptosis regulation (83). In obesity-related asthma models, adiponectin reduces airway inflammation and oxidative stress partly through AMPK (46), while AMPK constrains Smad-dependent profibrotic signaling and epithelial-to-mesenchymal transition (106). These observations connect adiponectin to epithelial protection through repair-supporting, antioxidant, and anti-profibrotic pathways. A decline in adiponectin may therefore increase susceptibility to oxidative epithelial injury and compromise orderly re-epithelialization. Adiponectin insufficiency is best positioned as an upstream injury-repair modifier, not a standalone remodeling endpoint, creating a vulnerable epithelial context for downstream programs.

Within this vulnerable epithelial context, leptin-related evidence helps define two downstream remodeling outputs. One output is secretory remodeling. In human bronchial epithelial cells, leptin enhances IL-13-induced MUC5AC production and secretion (32). In IL-33-driven models in leptin-deficient obese mice, endogenous leptin also supports goblet cell metaplasia and MUC5AC-related responses (84). Secretory lineage allocation can be assessed through differentiation programs such as Notch signaling, which skews human airway basal cells toward secretory cell fate (107, 108). The second output is fibrotic repair at the epithelial-subepithelial interface, linking epithelial plasticity to activation of the underlying mesenchymal. In airway epithelial cells from patients with asthma, TGF-β_1_ can induce epithelial-mesenchymal transition (109), and IL-1β further enhance this process in human bronchial epithelial cells (110). In parallel, leptin-rich conditions reinforce the mesenchymal side of this repair program, because exogenous leptin enhances peribronchiolar collagen deposition, lung fibroblast invasiveness, and Col1a1 expression in a chronic allergic airway disease model (76), and cross-disease lung fibroproliferative evidence indicates that leptin can augment TGF-β_1_-driven profibrotic gene transcription in cultured human lung fibroblasts by inhibiting PPARγ (111). In asthmatic airway epithelium, reduced leptin/LepR expression in association with increased reticular basement membrane thickness and epithelial profibrotic signaling supports local LepR attenuation as a tissue-level marker of the fibrotic remodeling interface (104).

Together, adipokine imbalance may promote aberrant epithelial repair by reducing adiponectin-AMPK protection, fostering leptin-associated secretory remodeling, and marking a TGF-β-related fibrotic interface through local LepR attenuation. Candidate airway readouts include mucus production, goblet cell metaplasia, secretory lineage allocation, LepR expression, profibrotic epithelial activity, epithelial repair markers, and basement-membrane thickness, stratified by obesity, disease duration, and remodeling severity.

### 5.4 Adipokine-driven ASM proliferation and phenotypic transition

The increase in airway smooth muscle mass that characterizes advanced asthmatic remodeling reflects both cellular hypertrophy and hyperplasia, and may contribute to less reversible airflow obstruction and persistent bronchial hyperreactivity in long-standing disease. ASM proliferation in asthma is regulated by intracellular mitogenic pathways including PI3K/Akt/mTOR and MAPK/ERK1/2, which integrate growth factor, cytokine, extracellular-matrix, and metabolic-inflammatory cues (112, 113). Airway smooth-muscle cells from asthmatic subjects proliferate more rapidly than cells from non-asthmatic subjects, and mitogen-driven ASM growth is controlled by ERK- and PI3K-dependent pathways with disease-specific signaling differences (114). Pharmacological targeting of PI3K/Akt/p70S6K in cultured human ASM cells reduces mitogen-stimulated cell number and cyclin D1 abundance, including in cells derived from asthmatic donors (115). Therefore, these studies identify PI3K/Akt/mTOR and MAPK/ERK signaling as core ASM growth pathways that may integrate metabolic-inflammatory inputs during airway remodeling.

Within this established mitogenic framework, adipokine dysregulation may influence ASM remodeling by altering growth-regulatory constraints rather than by acting as a uniform direct mitogenic stimulus. Evidence from adiponectin-treated obese asthma models links adiponectin to AMPK-mediated airway protection, while independent ASM experiments show that AMPK activation suppresses basal and TGF-β_1_-induced ASM proliferation (46, 116, 117), suggesting that adiponectin deficiency may weaken an AMPK-linked anti-proliferative constraint on ASM growth. By contrast, leptin appears more relevant as a context-dependent modulator of ASM-associated growth-factor and inflammatory responses than as an isolated mitogenic stimulus, because leptin promotes VEGF release but does not consistently stimulate human ASM proliferation (38). Together, these findings suggest that adipokine dysregulation may contribute to ASM remodeling by weakening adiponectin-AMPK anti-proliferative signaling and modifying mitogenic pathways, while leptin alone is unlikely to act as a consistent direct driver of ASM proliferation.

### 5.5 Adipokine-mediated fibroblast activation and ECM remodeling

Resident fibroblast conversion to an activated myofibroblast phenotype is a key effector of subepithelial fibrosis (118). This phenotype features α-smooth muscle actin (α-SMA) expression, enhanced contractility, and increased ECM production (119). Leptin receptor-mediated signaling can enhance inflammatory cytokine and chemokine production by lung fibroblasts (120). Adipocyte-fibroblast coculture further show that adipocytes from obese donors promote fibroblast-to-myofibroblast transition and collagen deposition through TGF-β_1_-centered pathways (100). These findings provide a cellular link between adiposity and fibroblast activation and support patient-derived adipocyte-fibroblast systems for modeling adipokine-rich airway microenvironments.

Once fibroblasts are activated, ECM remodeling can extend local adipokine-driven signaling into persistent structural change. Progressive matrix deposition within the subepithelial layer is thought to increase airway wall stiffness, reduce luminal compliance, and contribute to fixed airflow limitation in asthma remodeling (101, 118). ECM-associated proteins can also mark profibrotic airway remodeling, as illustrated by periostin induction downstream of IL-4/IL-13 signaling in bronchial asthma (121). Matrix burden is shaped by ECM turnover, with MMP-9 expression linked to subepithelial fibrosis in asthmatic airway inflammation and higher macrophage-derived MMP-9/TIMP-1 ratios associated with faster FEV1 decline in chronic asthma cohorts (122, 123). Increased matrix stiffness can activate YAP/TAZ-dependent mechanotransduction in lung fibroblasts and support myofibroblast formation (124, 125). In OBA, adipokine-rich inflammation may therefore advance remodeling by promoting fibroblast activation and ECM turnover. After matrix stiffness and mechanotransductive signaling are established, structural remodeling may become less dependent on the initiating metabolic-inflammatory stimulus.

Reversibility of adipokine-associated remodeling likely varies across disease stage and structural compartment. Early remodeling remains closely linked to active inflammation, epithelial injury, mucus hypersecretion, early fibroblast activation, and dynamic ECM turnover, making these components plausible targets for metabolic improvement or attenuation of leptin-dominant inflammatory signaling (101, 102, 118). In contrast, once structural remodeling is established, correction of systemic adipokine imbalance may attenuate further progression but is unlikely to fully reverse existing architectural alterations, including collagen maturation or cross-linking, established subepithelial fibrosis, persistent ASM hypertrophy or hyperplasia, matrix stiffening, and fixed airflow limitation (101, 102, 118, 126). At present, longitudinal evidence remains insufficient to determine whether sustained restoration of adiponectin-dominant signaling or leptin receptor responsiveness over months or years can fully reverse established cellular and extracellular remodeling in OBA. Current evidence also does not support a single irreversible threshold, but instead suggests a remodeling-plasticity spectrum in which reversibility progressively decreases as inflammation-dependent remodeling gives way to stable architectural change. This stage-dependent decline in remodeling plasticity highlights the importance of early metabolic intervention in OBA and underscores the need for structural endpoints to determine whether metabolic intervention slows remodeling progression, preserves residual plasticity, or modifies established disease across distinct airway compartments.

Taken together, the evidence reviewed above indicates that adipokine imbalance shape OBA through concurrent effects on airway inflammation, hyperresponsiveness, and remodeling. Observed links between adipokine levels and OBA features depend on whether signals are read systemically in the circulation or locally in sputum, BAL, airway tissue, or wall-adjacent adipose interfaces. Because these compartments provide complementary information, **Table 1** synthesizes systemic and local adipokine associations with OBA-related outcomes and their candidate mechanisms.

**Table 1:** Systemic versus local adipokine-OBA associations and candidate mechanisms.

Domain	Adipokine/readout	Compartment	OBA-related outcome(s)	Association (summary)	Candidate mechanism(s)	Refs
Inflammation	Leptin	Systemic (serum/plasma)	Asthma risk; obesity-linked inflammatory phenotype; neutrophilic or mixed inflammation	Often elevated in asthma/obesity contexts; modified by sex and phenotype	Macrophage inflammatory activation (obR/JAK-STAT); context-dependent neutrophil responses; Th2/ILC2 amplification in allergic OBA	(26, 31, 41, 64)
	Leptin	Local (sputum/BAL)	Airway inflammatory tone; obesity-related airway phenotype	usually interpreted with obesity status rather than as a standalone marker; airway levels poorly phenotype-linked beyond obesity status	Local immune-cell inflammatory programs driven by endocrine spillover into the airway milieu	(64, 127, 128)
	Adiponectin	Systemic	Adult obesity-dominant asthma risk/severity; anti-inflammatory tone	Often lower in adult/obesity-dominant contexts; pediatric patterns more heterogeneous	Loss of AdipoR/AMPK-linked restraint on macrophages and epithelium	(21, 26, 46–48)
	Adiponectin	Local (sputum/BAL)	Airway anti-inflammatory tone	Measurable but limited relative to systemic measures and sparsely phenotype-linked	Local AdipoR-AMPK signaling (largely inferred)	(127, 128)
	Resistin; resistin:adiponectin ratio	Systemic	Asthma risk; lung function (e.g., FEV_1_)	Risk associations reported; elevated ratio linked to lower FEV_1_ in some analyses; associations interpreted in light of sex, weight, and corticosteroid exposure	Pro-inflammatory adipokine excess (resistin↑) with reduced adiponectin restraint (elevated resistin:adiponectin ratio); pathway-level airway mapping limited relative to leptin/adiponectin	(13, 58)
AHR	Leptin/leptin resistance	Systemic ± neural axis	AHR; bronchomotor reactivity	Experimental support for AHR modulation; neural disinhibition under chronic obesity-related leptin resistance	Inflammatory amplification of AHR; parasympathetic/cholinergic tone via leptin signaling and resistance	(30, 38, 94)
	Adiponectin/AdipoR-AMPK	Systemic, with airway-relevant experimental support	AHR; bronchoconstrictive susceptibility	Protective signals in models; human ASM-focused validation still needed	AMPK-linked bronchoprotection; reduced constraint when adiponectin is low	(44, 46)
	Metabolic inflammasome axis in adipokine-enriched OBA	Systemic metabolic milieu to airway innate pathways	Obesity-linked or corticosteroid-insensitive AHR	Supported mainly by HFD/innate models rather than a single circulating adipokine assay	NLRP3/IL-1β-ILC3-IL-17A and related innate programs	(16, 85–89)
Remodeling	Airway-wall/PBAT-AAAT adipose	Local	Airway wall thickness; inflammatory-cell density; structural remodeling risk	Adipose area correlates with BMI, wall thickness, and inflammation in human large airways	Local paracrine adipose-airway crosstalk; fibroblast activation niche	(60)
	Leptin	Local (airway fibroblast/epithelial interface)	Chemokine milieu; fibrosis-related markers	Can enhance IL-13-linked eotaxin responses and fibrosis-related features in experimental systems	Fibroblast chemokine production; TGF-β-linked fibrotic programs	(75, 76)
	Leptin/LepR expression	Local (airway epithelium/tissue)	Reticular basement membrane thickness; epithelial profibrotic signaling	Reduced local leptin/LepR linked to thicker basement membrane and TGF-β-related features; attenuated LepR may mark a fibrotic epithelial–subepithelial interface	Preserved LepR-linked inflammatory amplification when signaling is intact; attenuated local LepR with TGF-β-related epithelial profibrotic programs and thicker basement membrane	(104)
	Adiponectin/AMPK	Systemic signal with local structural effectors	Epithelial repair failure; ASM growth constraint; progression toward fixed obstruction	Protective and anti-proliferative constraints weakened when adiponectin/AMPK tone is low	Epithelial repair and antioxidant support; AMPK restraint of ASM proliferation and profibrotic signaling	(46, 83, 116, 117)

## 6 Clinical implications and potential therapeutic strategies

### 6.1 Metabolic-airway phenotyping and biomarker stratification

The convergence of adipokine-associated inflammation, AHR, and remodeling supports the view that at least a subset of OBA represents a metabolically conditioned airway phenotype rather than the simple coexistence of obesity and asthma. In this phenotype, leptin/obR-driven macrophage activation may shape neutrophilic inflammatory tone (64), airway-associated adipose tissue is associated with airway wall structure and inflammatory-cell density (60), and systemic IL-6 marks a clinically severe metabolic-inflammatory subgroup (129). Together, these mechanisms may be relevant to corticosteroid responsiveness, exacerbation burden, airway structure, and progressive remodeling risk. Clinical assessment should therefore move beyond body mass index alone and incorporate markers of both metabolic dysfunction and airway disease activity.

Serum leptin and adiponectin, and in some studies the leptin-to-adiponectin ratio, have been proposed as candidate markers because they are associated with asthma risk, disease severity, or obesity-linked inflammatory activity. A meta-analysis supports overall asthma associations with leptin and adiponectin (26), while clinical studies and syntheses have examined leptin status, leptin-focused prognostic value, and the leptin-asthma-obesity link (130, 131). Their clinical value may also need to be interpreted alongside sex, age, adiposity distribution, insulin resistance, oral corticosteroid exposure, and developmental differences between pediatric and adult adipokine profiles (26, 48). Peripheral blood concentrations may not fully capture airway- or adiposity-distribution-related structural associations, as suggested by airway-wall adipose tissue and CT-based adiposity studies (60, 132).

Among clinically available measures of adiposity distribution, waist circumference (WC) is a simple, noninvasive surrogate of central adiposity that can be obtained routinely in the clinic and is therefore relevant when interpreting adipokine levels in OBA. Studies in asthma and overweight/obese cohorts have combined circulating leptin measurements with WC. In these investigations, WC is positively correlated with blood leptin, and leptin is a partial mediator of the association between WC-defined central adiposity and persistent asthma (14, 133, 134). In patients with asthma, blood adiponectin is usually lower when abdominal adiposity is higher, whereas WC associations for resistin and other adipokines remain limited (133, 135). Locally, leptin and adiponectin are measurable in BAL fluid or sputum in obesity-related asthma, yet these airway levels have usually been analyzed against BMI or obesity status rather than WC (75, 127, 128). In summary, some reports have combined adipokine measurements with WC in obese-asthma-related research, chiefly for systemic leptin; joint assessment of BAL/sputum adipokines with WC remains largely lacking.

Taken together, adipokines are better positioned as stratification markers than as independent diagnostic biomarkers. Their value is likely to be greatest when combined with measures of adiposity distribution, metabolic status, blood or sputum inflammatory profiles, lung function, exacerbation history, corticosteroid response, and structural airway assessment where available. Such integrated phenotyping could help identify patients whose asthma is strongly influenced by metabolic-inflammatory mechanisms and who may be more suitable for metabolic intervention or future pathway-directed trials.

### 6.2 Metabolic intervention and the importance of disease stage

Weight reduction remains one of the best-supported strategies for modifying the upstream metabolic context of OBA. Forno et al. reported that the impact of bariatric surgery on asthma control differed by metabolic syndrome status (136). van Huisstede et al. documented post-bariatric improvements in asthma control, lung function, and bronchial or systemic inflammatory measures in morbidly obese subjects with asthma (23). Dixon et al. further showed that bariatric surgery-associated improvement in AHR was observed only in participants with normal IgE levels, not in those with elevated IgE, suggesting that immune profiles of obese asthma patients influence outcomes following weight loss (22). These findings support the clinical relevance of metabolic dysfunction in asthma expression and provide a strong rationale for integrating metabolic and adipokine endpoints into metabolic intervention studies. Because weight loss modifies both mechanical load and metabolic-inflammatory tone, including adipokine balance, clinical gains should be read with adipokine changes rather than weight alone. In obese asthma, bariatric or intensive weight-loss cohorts show lower systemic leptin, higher adiponectin or adiponectin-to-leptin ratio, and better asthma control; restored adipokine balance has also been linked to improved corticosteroid responsiveness, and visceral adipose leptin expression correlates with AHR that improves after surgery (137–139). Few studies test whether leptin reduction or adiponectin recovery predicts FEV_1_, control, or exacerbations independent of weight loss. Airway (BAL/sputum) adipokine data after treatment also remain scarce, so integrating metabolic and adipokine endpoints into intervention studies is warranted.

GLP-1 receptor agonists extend this therapeutic concept into pharmacological metabolic intervention. In OBA models, GLP-1 receptor activation reduces airway inflammation and AHR, in part through suppression of NLRP3 inflammasome activity and IL-1β signaling (140). Clinical and real-world studies report asthma-relevant outcomes associated with GLP-1 receptor agonists, including improved asthma control, fewer exacerbation-related outcomes, or reduced asthma risk in selected obese populations (141–143). Related evidence from obesity-associated sleep-disordered breathing also shows that the dual GIP/GLP-1 receptor agonist tirzepatide can improve respiratory comorbidity outcomes in the SURMOUNT-OSA trial (144). Beyond these clinical signals, meta-analyses of randomized trials in metabolic populations show that GLP-1 receptor agonists lower circulating leptin and resistin and raise adiponectin (145, 146). Whether the effects of GLP-1-based weight-loss therapy on asthma-related outcomes in OBA are mediated by these adipokine changes remains to be established. Prospective GLP-1 receptor agonist studies in OBA should therefore include both asthma clinical endpoints and serial adipokine measurements.

Disease stage is likely to influence the magnitude and type of clinical benefit. In patients with inflammation predominant disease, bariatric-surgery cohorts suggest that metabolic intervention may reduce selected systemic or airway inflammatory measures, improve AHR or airway responsiveness, and enhance treatment control (22, 23). In patients with established remodeling, fixed airflow limitation, ECM deposition, ASM expansion, and persistent wall thickening may be less reversible based on general asthma remodeling literature (101, 102). Early metabolic intervention may therefore represent a testable disease-modifying window and should be evaluated with structural endpoints. Endpoint selection should also reflect disease stage. For instance, inflammatory biomarkers, adipokine dynamics, exacerbations, AHR, and corticosteroid responsiveness are most relevant for earlier disease, whereas imaging, oscillometry, small airway indices, and remodeling markers are more appropriate for structurally advanced disease.

### 6.3 Mechanism-enriched trial design and translational endpoints

The pathways reviewed above should currently be used as candidate enrichment and mechanistic-readout frameworks rather than as established therapeutic targets in OBA. Future trials should stratify participants by adipokine profile, adiposity distribution, metabolic comorbidity, T2 versus non-T2 inflammation, AHR, corticosteroid responsiveness, and remodeling status, while pairing clinical outcomes with pathway-matched endpoints such as JAK/STAT-linked immune activation, NLRP3/IL-1β/IL-17A-related innate inflammation, IL-6-associated systemic inflammatory burden, airway mechanics, and structural remodeling measures. Such designs could clarify whether pathway-directed interventions improve OBA through specific metabolic-inflammatory airway mechanisms rather than through nonspecific effects on weight or asthma control.

## 7 Conclusion and future perspectives

At least a subset of OBA should be viewed as a metabolically conditioned airway disease in which adipokine imbalance contributes to the heterogeneity of airway inflammation, hyperresponsiveness, and remodeling. Leptin excess and adiponectin insufficiency provide a biologically coherent link between obesity-related metabolic dysfunction and airway pathology, although the strength and directness of evidence vary across immune, functional, and structural domains.

A spatial temporal interpretation may help reconcile several unresolved issues in this field. Systemic adipokine concentrations capture the endocrine component of obesity-related inflammation, whereas airway-associated adipose tissue and PBAT may provide local paracrine interfaces that influence epithelial cells, ASM, fibroblasts, and resident immune cells. Over time, persistent metabolic inflammation may couple epithelial repair failure, receptor desensitization, fibroblast activation, and ECM deposition, potentially shifting disease expression from more reversible inflammatory dysfunction toward remodeling-predominant pathology.

Overall, the central challenge moving forward lie in transitioning from systemic associations to airway-localized mechanisms and from broad obesity-based classification to mechanism-informed OBA subgroups. This transition will be essential for determining when adipokine imbalance is a causal contributor, a disease modifier, or a stratification marker, and for identifying patients most likely to benefit from precision metabolic-airway interventions.
